# Noise facilitates entrainment of a population of uncoupled limit cycle oscillators

**DOI:** 10.1098/rsif.2022.0781

**Published:** 2023-01-11

**Authors:** Vojtech Kumpost, Lennart Hilbert, Ralf Mikut

**Affiliations:** ^1^ Institute for Automation and Applied Informatics, Karlsruhe Institute of Technology, Eggenstein-Leopoldshafen, Germany; ^2^ Institute of Biological and Chemical Systems—Biological Information Processing, Karlsruhe Institute of Technology, Eggenstein-Leopoldshafen, Germany; ^3^ Department of Systems Biology and Bioinformatics, Zoological Institute, Karlsruhe Institute of Technology, Karlsruhe, Germany

**Keywords:** circadian clock, cellular noise, stochastic oscillator

## Abstract

Many biological oscillators share two properties: they are subject to stochastic fluctuations (noise) and they must reliably adjust their period to changing environmental conditions (entrainment). While noise seems to distort the ability of single oscillators to entrain, in populations of uncoupled oscillators noise allows population-level entrainment for a wider range of input amplitudes and periods. Here, we investigate how this effect depends on the noise intensity and the number of oscillators in the population. We have found that, if a population consists of a sufficient number of oscillators, increasing noise intensity leads to faster entrainment after a phase change of the input signal (jet lag) and increases sensitivity to low-amplitude input signals.

## 1. Introduction

Many cellular processes show oscillatory behaviour. This includes circadian clocks [1], cardiac pacemakers [2] and various signalling proteins like p53 [3] and NF-*κ*B [4]. Although the rhythms generated by these systems manifest on different timescales and have different functions within the organism, in terms of a mathematical representation, they can be modelled as a limit cycle oscillator that consists of a negative feedback loop with a delay [5]. Furthermore, cellular oscillators constantly adjust their phase and period based on the changes in the environment, thereby achieving entrainment [6]. In this way, cellular processes can be reliably timed with respect to environmental conditions. Cellular processes, including oscillators, are frequently affected by noise stemming from a low number of reacting molecules involved in the feedback regulation [7]. In consequence, a low number of molecular interactions occur at stochastically distributed times, resulting in stochastic variations in the periodic gene expression. Although noise and entrainment are individually recognized to influence cellular oscillators, it remains unclear whether noise is generally detrimental to entrainment, or might in fact be co-opted in the entrainment of cellular oscillators.

The circadian clock is one of the most prominent biological oscillators and ensures the correct timing of various biological processes with respect to the time of the day [8]. The circadian clock in mammals is organized hierarchically with a central peacemaker of highly coupled oscillators in the suprachiasmatic nucleus (SCN) and weakly coupled or uncoupled oscillators in the peripheral tissues [9]. While a lot of research focused on the dynamics of the highly coupled SCN cells, the limited coupling in peripheral tissues results in distinctly different dynamics that might be better explained by an uncoupled population model. For example, the weaker coupling between peripheral clocks allows entrainment for a wider range of input periods [10] and additional external cues such as changes in ambient temperature [11]. The population of uncoupled oscillators is also relevant in the correct analysis of the circadian bioluminescence assays [12,13]. Here, a better understanding of the dynamics at the population level in the presence of noise would help in inferring the single-cell behaviour from the population-level recordings. For those reasons, it is of great interest to better understand the significance of noise in the entrainment of uncoupled stochastic oscillators.

The implications of noise on the population-level entrainment are, however, still poorly understood. In this study, we examine the effect of noise on the population-level entrainment of uncoupled stochastic oscillators for different population sizes (number of oscillators), noise intensities and amplitudes and periods of the input signal. Previous work found that noise allows population-level entrainment to a wider range of input amplitudes and periods than a single stochastic or deterministic oscillator [14]. We extend those findings by examining the change in the entrainment for varying population size and noise intensity. In addition, we examine how different levels of noise influence the response to a perturbing pulse under constant pacing conditions (phase response curve (PRC)) and the recovery from a phase shift in the input signal (jet lag). We have found that noise expands the range of input amplitudes and periods for which entrainment occurs, with an optimal noise intensity for a given number of oscillators in the population. In our simulations, noise also increases the response of the oscillator population to a perturbation and shortens jet lag. Finally, we used the canonical amplitude-phase and Van der Pol models, which represent the class of sinusoidal and relaxation limit cycle oscillators, respectively, to show that we come to the same conclusions. Thus these findings might be interesting not only for the modelling of cellular oscillators but also for the wider class of natural and bioinspired technical systems with uncoupled or loosely coupled oscillators under the influence of external triggers and noise.

## 2. Results

### 2.1. Model of a population of uncoupled stochastic oscillators

**Figure 1.**
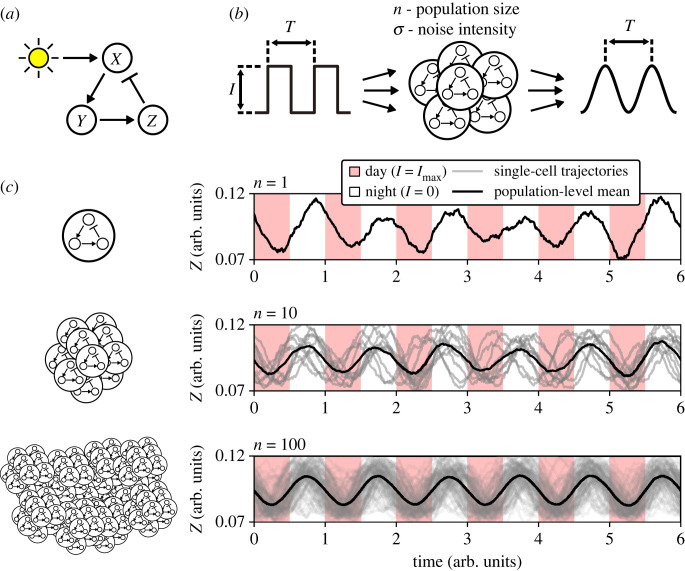
A model of population-level entrainment of uncoupled cellular oscillators. (*a*) A minimal oscillator model consists of three variables (*X*, *Y*, *Z*) connected in a negative feedback loop. Input, e.g. light in the case of the circadian clock, is implemented via addition to *X*. (*b*) The model from (*a*) represents a single-cell oscillator in a population. A number of oscillators are combined into a population that is driven by a square signal with period *T* and amplitude *I*. The input square signal switches between a high state and a low state, e.g. day and night of the day–night cycle. The output is calculated as the mean of the output of the individual oscillators and, if entrained, oscillates with the same period *T* as the input signal. We aim to explore the effect of changing population size (number of oscillators, *n*) and noise intensity (*σ*) on the population-level entrainment. (*c*) Example results of numerical simulations for a single-cell oscillator (*n* = 1) and two populations of different sizes (*n* = 10, 100). A higher population size results in a smoother periodic signal and thus reduces stochastic fluctuations.

We wish to examine how molecular noise affects the population-level entrainment of a population of uncoupled cellular oscillators. For this purpose, we use a minimal Kim–Forger model [15] with additive light input and multiplicative noise terms, which represent the molecular noise stemming from the discrete nature of cellular chemical interactions [16]. We have shown previously that such a simple structure captures the entrainment dynamics in a circadian bioluminescence reporter assay with high precision while reducing the number of adjustable model parameters [17]. The core model consists of three variables connected in a negative feedback loop and represents the dynamics of one cellular oscillator (figure 1*a*). This simplified Kim–Forger model (equation (4.1)) has one free parameter for which we assigned a fixed value to obtain a limit cycle oscillator with high-amplitude oscillations (electronic supplementary material, figure S1). For convenience, the model is time-scaled to oscillate with a unit period if noise terms and input signals are set to zero. To obtain a population-level output from the single-cell model, we repeat the simulation *n* times and calculate the mean over the individual trajectories, thereby representing an experimental recording from a population of uncoupled identical stochastic oscillators that are driven by the same input signal (figure 1*b*). An intuitive strategy to minimize the stochastic fluctuations at the population level is to increase the number of oscillators in the population (figure 1*c*). This approach illustrates that even a not obviously stochastic population recording stems from inherently stochastic oscillators. This stochasticity may influence the population behaviour, even if not apparent after averaging over the population of oscillators.

### 2.2. Noise widens range of entrainment in oscillator populations

**Figure 2.**
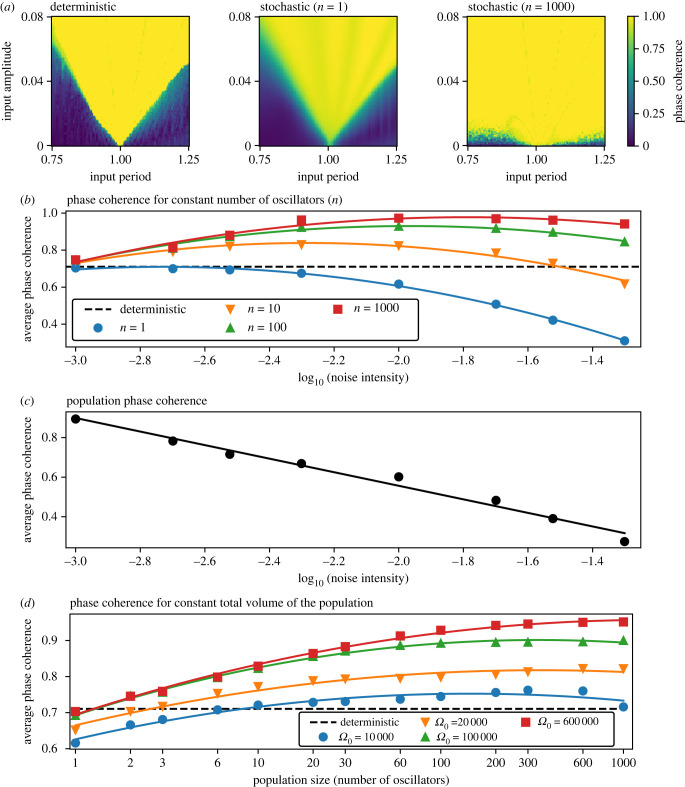
Noise widens the range of entrainment in oscillator populations. (*a*) Arnold tongue for a deterministic model (*σ* = 0) and a stochastic model (*σ* = 0.005) simulated with two different population sizes. The colour coding of the Arnold tongue displays PC that quantifies the quality of entrainment as a number between 0 (no entrainment) and 1 (full entrainment) (electronic supplementary material, figure S2). We quantified the Arnold tongues by their average value. In the example of the shown tongues, the average PC is 0.71, 0.67 and 0.96 for the deterministic, single-cell stochastic *n* = 1 and population stochastic *n* = 1000, respectively. (*b*) Dependence of the average PC on the noise intensity. The individual Arnold tongues used to generate this image are shown in electronic supplementary material, figure S3. Solid lines are second-order polynomial fits. (*c*) Dependence of the average population phase coherence (PPC) on the noise intensity. PPC quantifies the desynchronization of the individual oscillators in the population (electronic supplementary material, figure S4). The individual Arnold tongues used to generate this image are shown in electronic supplementary material, figure S5. (*d*) Dependence of the average PC on the increasing population size and constant total system size ($\Omega_{0}$). The individual Arnold tongues used to generate this image are shown in electronic supplementary material, figure S6. Solid lines represent second-order polynomial fits.

We used phase coherence (PC) as a continuous metric of entrainment of the oscillator population to the external periodic signal (equation (4.10), electronic supplementary material, figure S2). Arnold tongues visualize how this entrainment depends on the amplitude and period of the input signal [18,19]. In general, a greater amplitude of the input signal increases the range of periods for which the system is entrained (indicated by a PC close to 1), resulting in the typical, tongue-shaped regions of entrainment (figure 2*a*). It has been previously shown that if a population mean is considered, the Arnold tongue is wider in comparison with the Arnold tongue estimated with a single stochastic or deterministic model [14]. We were interested in how this widening of the entrainment range depends on the noise intensity and the number of oscillators used to construct the population mean. To quantify the Arnold tongues with a single number, we calculate the average PC of the tongue (figure 2*a*). Applying this metric to the output of a deterministic model (*σ* = 0) and a stochastic model (*σ* = 0.005), we found that the range of entrainment for the deterministic system is lower than for a stochastic population of 1000 oscillators (figure 2*a*). This is in line with previously published findings [14]. However, if we consider a single stochastic oscillator, the entrainment area drops to a lower value close to the deterministic model (figure 2*a*). This suggests that noise must be compensated for with a sufficiently large population size to allow the widening of the Arnold tongue by noise.

We extended this observation by measuring the entrainment area for four different population sizes and seven noise intensities (figure 2*b*, electronic supplementary material, figure S3). Using the deterministic case as a reference, we observed that for a single oscillator (*n* = 1) the range of entrainment decreases with increasing noise intensity, showing the detrimental effect of noise when only a single oscillator is considered. With increasing population size (*n* > 1), however, an optimal value of noise intensity exists, for which the range of entrainment is maximal. With increasing population size, this optimal noise intensity moves to higher values, and also the maximal range of entrainment at this optimum noise intensity is increased. Our results suggest an optimal noise intensity for the entrainment of a population of a given size. When this optimum noise intensity is exceeded, the entrainment capacity is again compromised.

To better understand how the observed population-level effect relates to the dynamics of the individual oscillators in the population, we used population phase coherence (PPC) as a metric of desynchronization among the individual oscillators within the population (equation (4.11), electronic supplementary material, figure S4). We found that with increasing noise intensity the PPC steadily decreases, indicating progressively more desynchronization among the individual oscillators (figure 2*c*, electronic supplementary material, figure S5). A moderate desynchronization of the individual oscillators thus supports the population-level entrainment; however, the amplitude of the population mean is progressively less prominent with increasing noise in comparison with the amplitude of the individual oscillators in the population.

To understand how a given population of cellular oscillators (cells) might be tuned towards optimal entrainment, we investigated a hypothetical case where a total volume *V* of cell material is available, but the number of cells (population size, *n*) and their individual system size (Ω) can change. In other words, we start with one big cell (Ω=V) that is subsequently divided into many small cells (Ω=V/n) while the total volume *V* remains constant. Here, the noise intensity (*σ*) depends on the system size of the individual cells (Ω) as $\sigma =1/\sqrt{\Omega }$. Our simulation results suggest an optimal population size for each total volume (figure 2*d*, electronic supplementary material, figure S6). Specifically, with increasing total volume, the maximal range of entrainment occurs at a higher population size and the average PC is also larger. In other words, a higher total volume can support a higher number of noisy oscillators, which in turn allows the population to take advantage of high noise intensities.

For completeness, we also simulated the possible effect of heterogeneity in the population on the population-level entrainment, thus assessing whether cell variability would have a similar effect as the intrinsic noise described above. In this experiment, we simulated a population of 1000 deterministic oscillators with varying values of the free parameter *A*. The parameter values for the population were drawn from a normal distribution with mean value *A*_0_ (default value of parameter *A*) and standard deviation *σ* (noise intensity). We found that the cell variability also increases the range of entrainment (electronic supplementary material, figures S7 and S8) similarly to the intrinsic noise with increasing noise intensity increasing also the range of entrainment.

### 2.3. Noise increases phase response to perturbations

**Figure 3.**
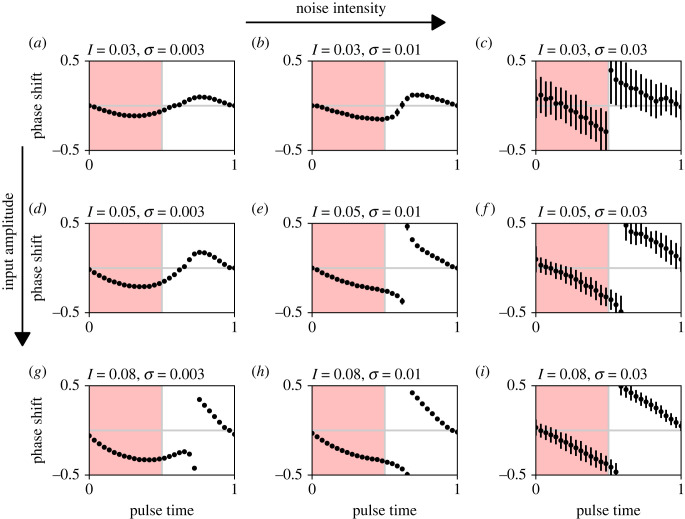
Phase response curves (PRCs) for varying noise intensity (*σ*) and input amplitude (*I*). Phase shift and pulse time are cyclic quantities normalized to the unit circle. The pulse length is 0.5, which is half of the free-running period for the deterministic model (*σ* = 0). The dots represent the mean and error bars the standard deviation over 10 simulations.

We also assessed whether noise can change the responsiveness of the population of uncoupled oscillators to perturbations. This can be explored using PRCs that plot the change in the oscillator phase caused by a step pulse as a function of the time during the oscillation cycle at which the pulse was applied [20,21]. In circadian research, the PRCs are often characterized based on their amplitude, which represents the extent of the pulse-induced phase shift, as type 1 or type 0. Type 1 PRCs exhibit relatively small phase shifts and appear continuous in the PRC plot, whereas type 0 PRCs show large phase shifts and appear visually discontinuous [20,21]. We have found that the PRC amplitude increases markedly not only with the increasing amplitude of the input signal but also with increasing noise intensity (figure 3). Specifically, for low input amplitude and low noise intensity, we observed relatively small phase shifts (type 1 PRC, figure 3*a*,*b*,*d*). As expected, when we increased the input amplitude, the phase shifts also increased. Interestingly, we observed the same effect also by increasing the noise intensity. For high noise intensities, we observed large phase shifts (type 0 PRC) regardless of the amplitude of the input signal (figure 3*c*,*f*,*h*). Accordingly, increasing noise intensity allows the transition from low-amplitude (type 1) to high-amplitude (type 0) PRC, even if the input amplitude remains low.

### 2.4. Noise allows faster recovery after jet lag

**Figure 4.**
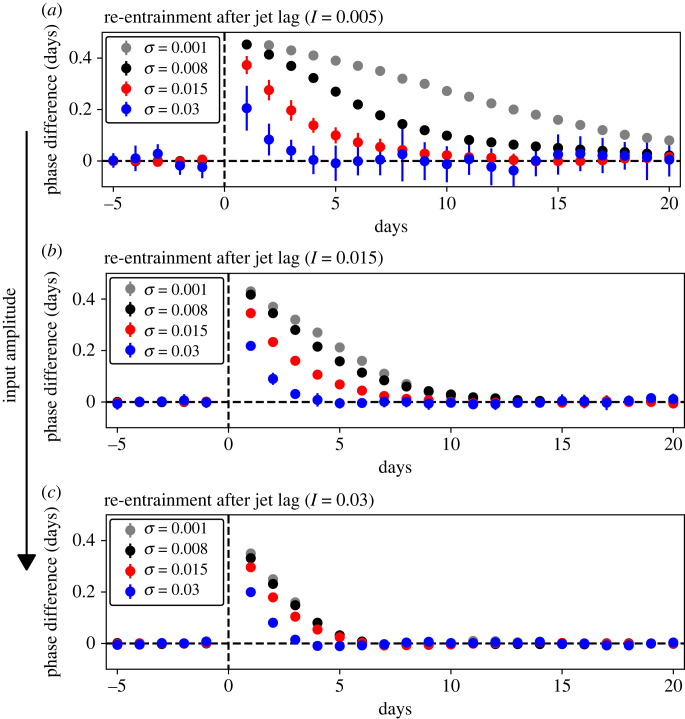
Increasing noise intensity (*σ*) allows faster re-entrainment after jet lag, especially for low input amplitudes (*I*). The phase difference indicates the difference between the phase on a specific day and the average phase of the days before the jet lag (electronic supplementary material, figure S9). Dots represent the mean and error bars the standard deviation over 10 simulations for all panels.

We also explored the effect of a varying noise intensity on the re-entrainment to a persistent phase shift in the input signal, or ‘recovery from jet lag’ (figure 4). In these simulations, the population is first entrained by a regular input cycle representing a day–night cycle. After the output of the population is phase-locked to the input signal, an abrupt shift in the phase of the input signal is introduced, and the time until the population output is locked to the new cycle is measured (electronic supplementary material, figure S9). We found that noise shortens this time, allowing faster recovery from jet lag. This is well visible when the input amplitude is low (figure 4*a*). With increasing input amplitude (figure 4*b*,*c*) re-entrainment to the new cycle is fast for all noise intensities, so the effect of the increasing noise is not as apparent. Accordingly, noise allows faster recovery from jet lag, especially if the input amplitude is low.

### 2.5. Noise facilitates the entrainment of limit cycle oscillators, but not of noise-induced oscillators

**Figure 5.**
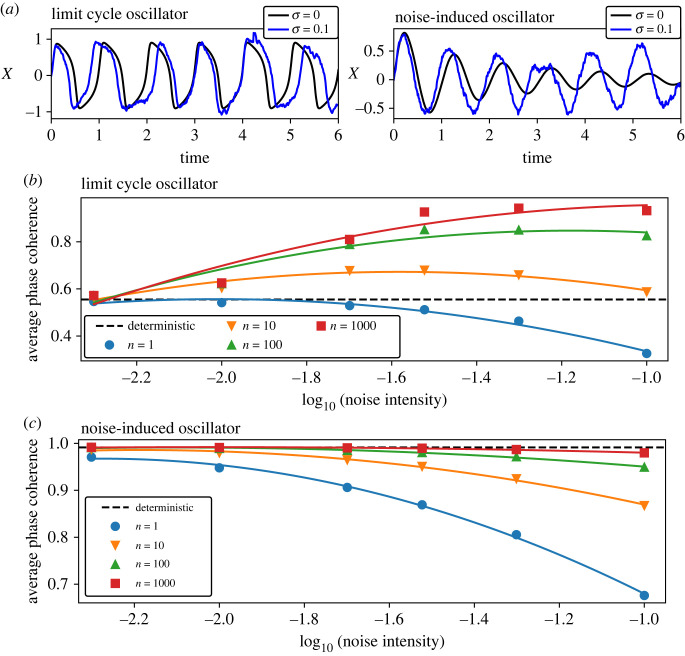
Noise widens the entrainment area for a limit cycle but not for a noise-induced oscillator. (*a*) Single oscillator example traces comparing deterministic (*σ* = 0) and stochastic (*σ* = 0.2) simulations for the Van der Pol model with parameters corresponding to a limit cycle and noise-induced oscillators. (*b*,*c*) Dependence of the average PC on the noise intensity for a limit cycle (*b*) and noise-induced (*c*) oscillator. The individual Arnold tongues used to generate those images are shown in electronic supplementary material, figures S10 and S13. Solid lines represent second-order polynomial fits.

To verify that the observed phenomena are not only a property of a negative feedback cellular oscillator but rather a general property of a limit cycle oscillator, we repeated all our experiments with the canonical Van der Pol model [22]. The Van der Pol model is a prototypic abstraction for limit cycle oscillators and is used in various fields in science and engineering [23], including biological oscillators such as circadian clocks [24] and cardiac pacemakers [25]. We used the Van der Pol model with additive input, noise terms and parameters as used previously to study the entrainment of a stochastic oscillator [26]. We explored the behaviour of the Van der Pol model under two different parameter sets, one corresponding to a limit cycle oscillator and one corresponding to a noise-induced oscillator (figure 5*a*). The noise-induced oscillator is a damped oscillator that generates sustained rhythms only in the presence of noise [27,28]. We found that the entrainment dynamics for the limit cycle model are equivalent to the results obtained with the Kim–Forger model in the previous parts of the manuscript. In particular, with increasing noise intensity and sufficient population size we can achieve enlarged Arnold tongues (figure 5*b*, electronic supplementary material, figure S10), increased amplitudes in PRCs (electronic supplementary material, figure S11) and faster recovery from jet lag (electronic supplementary material, figure S12A). By contrast to the limit cycle oscillator, the noise-induced oscillator showed wide Arnold tongues (figure 5*c*, electronic supplementary material, figure S13), high-amplitude PRCs (electronic supplementary material, figure S14) and short jet lags (electronic supplementary material, figure S12B) already for the deterministic model with zero noise intensity.

Limit cycle oscillators can be classified as relaxation oscillators, which generate non-sinusoidal oscillations, and sinusoidal oscillators, which have a harmonic limit cycle (electronic supplementary material, figure S15). This distinction is important in the study of entrainment as different classes of oscillators react differently to external forcing. For example, relaxation oscillators allow for faster entrainment [29]. We have already explored the behaviour of the relaxation limit cycle oscillator using the Van der Pol model above and were interested if the same results can also be achieved with the amplitude-phase model [10], a generic model for sinusoidal limit cycle oscillations (electronic supplementary material, figure S15). Repeating all assessments also for this model, we arrived at the same results, including increased average PC and enlarged Arnold tongues (electronic supplementary material, figures S16 and S17), increased amplitude in PRCs (electronic supplementary material, figure S18) and faster recovery from jet lag (electronic supplementary material, figure S12C). We thus conclude that the observations made in this study also work for a broader class of limit cycle oscillators that are subject to Gaussian noise but do not apply to noise-induced oscillators that generate only damped oscillations in the deterministic case.

## 3. Discussion

In this work, we explored the entrainment of a population of uncoupled stochastic oscillators represented by a minimal model of the circadian clock. We found that noise allows for population-level entrainment to a wider range of input signal periods and amplitudes. Noise also facilitates a larger response to external stimuli and faster recovery from jet lag. These effects emerge specifically at the population level, and cannot be observed in single oscillators. We used the canonical amplitude-phase and Van der Pol models to show that this behaviour emerges also for generic limit cycle oscillators, but not for noise-induced oscillators without a deterministic limit cycle. In the field of cellular oscillators and especially circadian clocks, these findings should contribute to a better understanding of the population behaviour of cells, for example in cell cultures, and how the population behaviour relates to the behaviour of single cells. Since our results seem to apply to various limit cycle oscillator systems, the results might not be limited to cellular oscillators but could be potentially applied to any domain where a population of noisy oscillators is under periodic pacing.

The results described in this work are the consequence of averaging the signals from the individual stochastic oscillators, which have a deterministic limit cycle and lack coupling. The results are thus mainly relevant for systems where only the average behaviour is of importance and synchronization among the individual cells is not crucial. Prominent examples of such systems are peripheral circadian clocks [9] and NF-κB signalling [30]. We observed that increasing noise intensity decreases the coherence of the individual oscillators but increases the population-level response to the changes in the input signal. The desynchronization within the population has, however, the consequence of decreasing the population-level oscillation amplitude in reference to the amplitude of the individual oscillators. We observed this effect for the stochasticity in the sense of the chemical master equation (Kim–Forger model) as well as for general sinusoidal (amplitude-phase model) and relaxation (Van der Pol) oscillator models with generic additive Gaussian noise terms. Therefore, we would expect similar effects also in other oscillator systems with uncoupled or loosely coupled oscillators with independent noise sources.

Previous work has shown that noise widens the population-level range of entrainment [14]. However, it has also been shown that, in the case of a single stochastic oscillator, the range of entrainment decreases with increasing noise intensity [31]. We have extended those findings by exploring the entrainment for a number of population sizes and noise intensities and found that for each population size, an optimal noise intensity exists. This existence of an optimal noise intensity is a typical characteristic of a phenomenon known as stochastic resonance [32]. The term stochastic resonance is traditionally used in neuroscience to describe improved detection of weak signals in threshold-like systems [33]. The term stochastic resonance is also used more generally to describe improvement in output performance of a noisy system in various disciplines including cell biology, ecology and physics [34]. In our system, noise improves the population-level entrainment but only to the point where the population size is sufficiently large to compensate for the noise-induced fluctuations at the population-level read-out. Thus, in comparison with the previous studies, we showed not only that noise widens the range of entrainment, but also that there exists an optimal value of the noise intensity, for which the range of entrainment is the widest.

The noise intensity (*σ*) is inversely related to the system size parameter that represents the number of interacting molecules (Ω): lower Ω gives higher *σ* and vice versa. We took advantage of this physical interpretation and perform a series of experiments, where the total number of molecules (*V*) is fixed and divided equally among *n* cells. We found that there is an optimal number of cells, for which the range of entrainment is the widest. In a general system, we could interpret *V* as the total amount of available resources or as a total price of a system and Ω as the number of resources taken by a single constituent unit of that system. We showed that, if the goal is maximal sensitivity in entrainment to input signals, it is more advantageous to distribute the resources to several noisy units rather than maintain a single unit with minimal noise. However, when the population size is increased beyond the optimum, the individual units become too noisy and the ability of entrainment is again compromised.

Biological cells are not only subject to intrinsic molecular noise, but also to external noise that stems from the heterogeneity of the population [35,36]. We implemented cell variability by varying parameter values of the individual oscillators and observed that population heterogeneity also leads to a wider range of population-level entrainment. This corresponds to the previous observations that cell variability allows the population to entrain robustly under a wider range of inputs [30]. However, cell variability also leads to a plethora of dynamic behaviours that is beyond the focus of this paper. The individual cells can exhibit widely different waveforms and some might even cross the Hopf bifurcation or exhibit chaotic behaviour [37], which presents a substantial challenge in the correct interpretation of the results. Therefore, our results on the cell variability should be considered rather preliminary and will be developed in detail in our future work.

In this work, we showed that the amplitude of the PRCs and speed of recovery from jet lag depends not only on the amplitude of the input signal, as described previously [38], but also on the intensity of the intrinsic noise. In our experiments, higher noise intensities led to higher PRC amplitude and shorter jet lags. These results might be particularly interesting in the context of the entrainment dynamics of the circadian clock. In mammals, the clock is thought to be organized hierarchically, with a central pacemaker of highly coupled oscillators in the SCN and less coupled peripheral oscillators that are entrained by signals from the SCN [9]. Pharmacologically increased noise in the SCN cells shortens jet lag even without explicitly weakening coupling among the cells [39]. This hints at the possible relevance of our results also to the domain of coupled oscillators. The coupling also provides the SCN with a certain level of resistance to noise and external perturbation [10,11]. Our results show that for increasing noise intensities the population becomes very sensitive to the input signals. This property would not seem useful to the SCN in maintaining a steady rhythm but might be advantageous for peripheral clocks that need to adjust to a manifold of external cues.

The positive effect of noise on the circadian clock is usually illustrated by noise-induced oscillations in the vicinity of a Hopf bifurcation [27,28]. From circadian data, however, it is often impossible to infer whether the circadian rhythms are formed by limit cycle or noise-induced oscillators [40]. It has been shown that a noise-induced oscillator can be entrained to a wider range of input amplitudes than a limit cycle oscillator [41]. We studied a general Van der Pol model with additive light and noise terms and with two parameter sets representing limit cycle and noise-induced oscillations [26]. Our results showed that, indeed, noise-induced oscillators entrain easier than limit cycle oscillators, not only at the single-cell level but also at the population level. However, the speed and range of entrainment for the limit cycle oscillator improve with increasing noise, whereas the entrainment properties of the noise-induced oscillator remain unchanged. For high noise intensities and sufficient population sizes, the entrainment dynamics of the limit cycle and noise-induced oscillators in fact become increasingly similar in terms of the wide range of entrainment, high-amplitude PRCs and short jet lags.

Another important application of our results is in inferring the single-cell dynamics from the population-level bioluminescence assays. These assays are relatively cheap and fast to conduct and lend themselves well to high-throughput screening of chemical compounds and mutants [42]. It has been shown that the cells in the bioluminescence assays behave as uncoupled oscillators [12,13]. Bioluminescence assays record a population mean over thousands of cells, so caution is required in making conclusions about single-cell behaviour. A common example of a discrepancy between the population-level and single-cell behaviour is the desynchronization of the cells in constant darkness that lead to the absence of oscillations at the population level, even though single-cell oscillations persist [43]. In the context of circadian entrainment, assays based on zebrafish cell lines are especially suitable because zebrafish cells are directly light-responsive [44]. These assays were also previously modelled as uncoupled oscillators, reproducing even detailed aspects of the experimental data [17]. Considering that noise can be pharmacologically enhanced in cell cultures [45], zebrafish assays might be a suitable experimental system to investigate some of the presented results in further *in vitro* experiments.

## 4. Methods

### 4.1. Mathematical models

We used three limit cycle oscillator models to explore the effects of noise on population-level entrainment: the Kim–Forger model as a model of a biological negative feedback oscillator, the Van der Pol model as a generic model of relaxation oscillations and the amplitude-phase model as a model of sinusoidal oscillations. Additionally, to also quantify the necessity of the existing limit cycle on the observed phenomena, we explored the Van der Pol model in an additional regime where it behaves as a damped oscillator.

#### 4.1.1. Kim–Forger model

The scaled Kim–Forger model (see electronic supplementary material for derivation) reads [15,46]

4.1a $$\frac{dX}{dt}=\;f(Z,A)-X+I,$$

4.1b $$\frac{dY}{dt}=X-Y,$$

4.1c $$\frac{dZ}{dt}=Y-Z$$

4.1d $$\begin{array}{rl} \mathrm{and}\;\;\;\;f(Z,A) & =\left\{ \begin{array}{cc} 1-\frac{Z}{A}\; & \frac{Z}{A}\leq 1\;\\ 0\; & \frac{Z}{A}>1\; \end{array}\right. \end{array},$$

where *X*, *Y*, *Z* are the concentrations, *A* is the free parameter and *I* is the input signal. Considering that each term in equation (4.1) represents a chemical reaction, we can construct a chemical reaction model as

4.2 $$\begin{array}{cccc} \text{no.} & \text{reaction} & \text{transition} & \text{transition rate}\\ 1 & \to x & x\to x+1 & \Omega \mathrm{kfr}(z,\Omega A)\\ 2 & x\to & x\to x-1 & x\\ 3 & \to x & x\to x+1 & \Omega I\\ 4 & \to y & y\to y+1 & x\\ 5 & y\to & y\to y-1 & y\\ 6 & \to z & z\to z+1 & y\\ 7 & z\to & z\to z-1 & z \end{array}$$

where *x*, *y*, *z* are the numbers of molecules and Ω is the system size parameter that determines the overall number of molecules in the system [47–49]. The number of molecules thus relate to the concentrations as x=ΩX, y=ΩY, z=ΩZ. As equation (4.2) is expressed in molecular numbers, and not concentrations as equation (4.1), also the transition rates must be scaled by the system size parameter Ω. This also applies to parameter *A* that has units of concentration and must be thus scaled by Ω as well.

The chemical reaction model in equation (4.2) can be simulated using the exact Gillespie method [50,51] that provides a correct solution for the chemical master equation. The Gillespie method is, however, computationally expensive for the increasing system size parameter Ω (electronic supplementary material, figure S19). For sufficiently large Ω, the discrete model can be approximated by the chemical Langevin equation [16] as

4.3a $$\frac{dX}{dt}=\;f(Z,A)-X+I+\sigma \left(\sqrt{\;f(Z,A)}\xi_{1}-\sqrt{X}\xi_{2}+\sqrt{I}\xi_{3}\right),$$

4.3b $$\frac{dY}{dt}=X-Y+\sigma \left(\sqrt{X}\xi_{4}-\sqrt{Y}\xi_{5}\right)$$

4.3c $$\mathrm{and}\;\frac{dZ}{dt}=Y-Z+\sigma \left(\sqrt{Y}\xi_{6}-\sqrt{Z}\xi_{7}\right),$$

where *X*, *Y*, *Z* are the molecular concentrations, *ξ*_*i*_ are the independent Wiener processes and *σ* is the noise intensity that scales with the system size as [27]

4.4 $$\sigma =\frac{1}{\sqrt{\Omega }}.$$

The stochastic differential equation (SDE) model was simulated using the Euler–Maruyama method with an integration step d*t* = 0.001. The integration step is sufficiently low to give comparable results with an accurate deterministic adaptive method for *σ* = 0 (electronic supplementary material, figure S20) as well as the exact Gillespie method for higher values of *σ* (electronic supplementary material, figure S21). The main disadvantage of the chemical Langevin equation is that for high noise intensities the concentrations might reach negative values, which make the evaluation of the terms under the square roots impossible in the real domain [52]. In our equation this problem relates mainly to the variable *X*, which oscillates close to 0 around the value given by the free parameter *A*. We thus set *A* = 0.1, which is high enough for *X* not to hit 0 also for moderate levels of noise but still low enough not to give the limit cycle (electronic supplementary material, figure S1). For this value of *A*, the chemical Langevin equation starts to break down for system size Ω<1000 (electronic supplementary material, figure S19). For those values of Ω, the Gillespie method was used as a fallback.

#### 4.1.2. Van der Pol model

As a generic model of a relaxation oscillator, we used the Van der Pol model in the form with external input and noise as used previously to study the entrainment of a stochastic oscillator [26]

4.5a $$\frac{dX}{dt}=Y+\sigma \xi_{1}$$

and

4.5b $$\frac{dY}{dt}=-(BX^{2}-d)Y-X+I+\sigma \xi_{2},$$

where *σ* is the noise intensity, *ξ*_*i*_ are the independent Wiener processes, *I* is the input and *d* and *B* are the free parameters that we set according to the previous study [26] to represent a relaxation limit cycle oscillator (*d* = 2, *B* = 10) and a noise-driven oscillator (*d* = −0.1 and *B* = 1). This model, including its parameters, was adapted from a previous study on the entrainment of stochastic oscillators [26]. The Van der Pol model is a generic model whose equations do not have a direct biological interpretation. Therefore, the noise terms are purely additive and represent a general stochastic disturbance rather than specifically molecular noise as in the Kim–Forger model. The SDE model was simulated using the Euler–Maruyama method with an integration step d*t* = 0.001.

#### 4.1.3. Amplitude-phase model

As a generic model of an oscillator with sinusoidal oscillations, we used the amplitude-phase model [53]

4.6a $$\dot{X}=\lambda X(A-R)-\omega Y+I+\sigma \xi_{1},$$

4.6b $$\dot{Y}=\lambda Y(A-R)+\omega X+\sigma \xi_{2}$$

4.6c $$\mathrm{and}\;\;\;\;\;R=\sqrt{X^{2}+Y^{2}},$$

where *σ* is the noise intensity, *ξ*_*i*_ are the independent Wiener processes, *I* the is input, *λ* is the time rate of the return to the limit cycle, *A* is the amplitude and *ω* is the angular frequency. The free parameters we set *λ* = *A* = *ω* = 1 according to the previous study [10]. Similar to the Van der Pol model, the amplitude-phase model is a generic model whose equations do not have a direct biological interpretation. Therefore, the noise terms are purely additive and represent a general stochastic disturbance, rather than specifically molecular noise as in the Kim–Forger model. The SDE model was simulated using the Euler–Maruyama method with an integration step d*t* = 0.001.

### 4.2. Model input

As the model input, we consider a square function

4.7 $$I=\left\{ \begin{array}{cc} I_{\max}\; & 0\leq (t\;\text{mod}\;T)<\frac{T}{2}\;\\ 0\; & \frac{T}{2}\leq (t\;\text{mod}\;T)<T\; \end{array}\right.,$$

where *T* is the input period and *I*_max_ is the input amplitude.

### 4.3. Model output

A population of uncoupled oscillators was simulated by calculating a mean of repeated independent numerical simulations as

4.8 $$X=\frac{1}{n}\sum_{i=1}^{n}X_{i},$$

where *n* is the number of oscillators in the population.

### 4.4. Metrics of entrainment

The complex-valued order parameter for a population of oscillators is defined as [54,55]

4.9 $$r\cdot e^{i\psi }=\frac{1}{n}\sum_{\;j=1}^{n}e^{i\phi_{j}},$$

where *r* is the degree of coherence, *ψ* is the collective phase, *ϕ*_*j*_ is the phase of the individual oscillators in the population and *n* is the number of oscillators in the population. The degree of coherence *r* is a number between 0 and 1, where *r* = 1 indicates that all oscillators are in the same phase and *r* = 0 indicates complete incoherence when the individual oscillators average out at the population-level mean. Based on the order parameter, we defined two metrics of entrainment: PC and PPC.

PC quantifies the population-level entrainment after the individual trajectories were averaged

4.10 $$\mathrm{PC}=\left|\frac{1}{N}\sum_{k=1}^{N}\;e^{i\varphi_{k}}\right|,$$

where *N* is the number of periods of the input signal, and φ_*k*_ is the time location of the population-level peak in *k*th cycle of the input signal. The PC ranges from 0 to 1. A value of 1 means that the peak of the population-averaged signal occurs exactly at the same time in every cycle. Values close to 0 indicate an unentrained signal with peaks occurring randomly within the cycle (electronic supplementary material, figure S2).

PPC quantifies the desynchronization of the individual oscillators in the population and is equivalent to *r* from equation (4.9) as

4.11 $$\mathrm{PPC}=\left|\frac{1}{n}\sum_{\;j=1}^{n}\;e^{i\phi_{j}}\right|,$$

where *n* is the number of oscillators in the population, and *ϕ*_*j*_ is the time location of the peak for *j*th oscillator in the population. The precision of this metric can be increased by calculating the circular mean of the population phase coherence over several periods of the input signal. The final value ranges from 0 to 1. A value of 1 indicates that peaks of all individual oscillators occur at the same time within one input cycle. Values close to 0 indicate a highly disperse population where each oscillator peaks at a different time of a cycle (electronic supplementary material, figure S4).

## Data Availability

Data and algorithms are available in a GitHub repository: https://github.com/vkumpost/stoosc. The data are provided in electronic supplementary material [56].
